# Draft Genome Sequence of Bacillus safensis Strain JG-B5T, Isolated from a Uranium Mining Waste Pile

**DOI:** 10.1128/MRA.00961-18

**Published:** 2018-09-13

**Authors:** Sarah Fischer, Thomas Krause, Norbert Jordan, Franziska Lederer, Rohan Jain

**Affiliations:** a Institute of Resource Ecology, Helmholtz-Zentrum Dresden-Rossendorf, Dresden, Germany; b Institute of Microbiology, Chair of Molecular Biotechnology, Technische Universität Dresden, Tatzberg, Dresden, Germany; c Helmholtz Institute Freiberg for Resource Technology, Helmholtz-Zentrum Dresden-Rossendorf, Dresden, Germany; Louisiana State University

## Abstract

Bacillus safensis strain JG-B5T was isolated from soil of the uranium mining waste pile Haberland located near Johanngeorgenstadt, Saxony, Germany. We report here a draft genome sequence (3.7 Mb) of this bacterial strain. The high metal resistance abilities of B. safensis strain JG-B5T can be exploited for bioremediation of metal and metalloid-contaminated environments.

## Abstract (precis)

Bacillus safensis strain JG-B5T was isolated from soil of the uranium mining waste pile Haberland located near Johanngeorgenstadt, Saxony, Germany. We report here a draft genome sequence (3.7 Mb) of this bacterial strain.

## ANNOUNCEMENT

The rod-shaped strain Bacillus safensis JG-B5T is an aerobic, spore-forming, Gram-positive soil bacterium and is observed to be a potent plant growth-promoting rhizobacterium (1). This bacterial strain colonizes a wide range of habitats, is able to survive in extreme environments, and has a high tolerance for salt and heavy metals, as well as UV and gamma radiation (2). The most famous type strain of this species, FO-36b^T^, was isolated from the spacecraft assembly facility of the Jet Propulsion Laboratory in Pasadena, California, USA (3).

B. safensis strain JG-B5T was isolated from the uranium mining waste pile Haberland near Johanngeorgenstadt, Saxony, Germany, in 1997 (4, 5) from soil obtained at a depth of 1 to 2 m and with a pH level of 4 (6).

The pure culture of B. safensis strain JG-B5T was routinely cultivated in nutrient broth (10 g/liter; Mast Group Ltd., Merseyside, UK) at 30°C. Genomic DNA of B. safensis strain JG-B5T was extracted using the MasterPure Gram-positive DNA purification kit (Epicentre) according to the manufacturer’s instructions. Purity and concentration of the DNA were determined using the NanoDrop 2000/2000c UV/Vis spectrophotometer (Thermo Scientific) and agarose gel electrophoresis (7).

AROS Applied Biotechnology A/S (Eurofins Genomics, Denmark) performed whole-genome sequencing of 200 ng of double-stranded DNA using next-generation sequencing technology with the Illumina HiSeq 2000 platform; 100-bp paired-end reads were used for whole-genome sequencing with a run time of 8 days. *De novo* assembly was performed using CLC Genomics Workbench software (Qiagen, Hilden, Germany). Genome annotation was done by the NCBI Prokaryotic Genome Annotation Pipeline (PGAP) (8). CheckM was used to calculate the completeness and purity of the reads (9).

The *de novo* assembly resulted in 16 contigs comprising a total of 3,657,238 bp (99.6% completeness and 0.4% impurity, estimated using CheckM), with an *N*_50_ value of 876,691 bp and a GC content of 42.8%. Average read coverage was 4,500× (calculated with CLC Genomics Workbench). The PGAP identified 3,710 protein-coding sequences and 64 RNAs, including 57 tRNAs; 16S and 23S rRNA sequence comparisons revealed B. safensis strains FO-36b, BRM1, U17-1, and U41 to be the most closely related, each sharing 100% sequence identity with strain JG-B5T.

The genome of B. safensis strain JG-B5T, isolated from a uranium mining waste pile, contains resistance determinants to various metals and metalloids, such as the arsenic efflux pump membrane protein ArsB, the cadmium-translocating P-type ATPase, the magnesium and cobalt transport protein CorA, and several heavy metal-translocating P-type ATPases. This organism has the potential to bioremediate metal- and metalloid-contaminated environments.

### Data availability.

This whole-genome shotgun project has been deposited at DDBJ/ENA/GenBank under the accession number QMDK00000000. The version described in this paper is the first version, QMDK01000000.
